# Adaptive Event-Triggered Distributed Estimation for a Class of Non-Linear Systems over Sensor Networks with Replay Attacks: The Finite-Horizon Case

**DOI:** 10.3390/e28080859

**Published:** 2026-08-01

**Authors:** Xianye Bu, Tao Lu, Wenbo Dong, Jiahui Li, Nan Hou

**Affiliations:** 1 School of Electrical and Information Engineering, Northeast Petroleum University, Daqing 163318, China; 2 Artificial Intelligence Energy Research Institute, Northeast Petroleum University, Daqing 163318, China

**Keywords:** distributed estimation, replay attacks, adaptive event-triggered, switching topologies

## Abstract

This work investigates the adaptive event-triggered distributed estimation problem for discrete time-varying nonlinear stochastic systems over sensor networks exposed to replay attacks within a finite-horizon setting. The sensor network comprises multiple nodes whose interaction structure is described by two randomly switching directed graphs. The plant under consideration is formulated as a discrete time-varying nonlinear stochastic system obeying a sector-bounded condition. To mitigate communication overhead, an adaptive event-triggered scheme is employed, where the triggering threshold is dynamically updated based on the triggering error. In addition, replay attacks are considered, wherein an adversary randomly replaces current data packets with previously recorded ones. A compensation mechanism is devised to neutralize the impact of such attacks. By building a distributed estimator and formulating an augmented estimation error system, sufficient criteria are established via Lyapunov theory and stochastic analysis to ensure the prescribed average $H_{\infty }$ performance level is attained. The estimator gains are computed recursively by solving a sequence of recursive linear matrix inequalities (RLMIs). A design algorithm for the distributed estimator is also provided to support online implementation. Finally, a numerical simulation example is given to demonstrate the effectiveness of the proposed estimation approach.

## 1. Introduction

Over the past few decades, the widespread adoption of networked systems in modern industry and smart applications has placed a growing emphasis on their performance and reliability. Wireless sensor networks (WSNs) have garnered considerable research interest worldwide owing to their promising applicability across a wide range of vital domains. This is particularly true in scenarios involving the detection of dynamic objects or operation in harsh environments, where wired sensor networks are impractical due to cabling constraints. In such contexts, WSNs offer a highly advantageous solution; see, e.g., refs. [1,2,3,4,5]. They demonstrate considerable promise across a wide range of applications, including urban traffic management, environmental monitoring in remote areas, disaster rescue operations, and security and information sensing in industrial parks. With the advancement of technology, the research on distributed filtering/estimation problems based on wireless sensor networks has received widespread attention from many scientists and has yielded fruitful results; see, e.g., refs. [6,7,8,9,10,11,12]. The distributed event-triggered set-membership filtering issue was investigated in [6] for sensor networks, where the nonlinear systems were subject to sensor saturation. The distributed $H_{\infty }$ filtering issue for discrete-time Takagi–Sugeno fuzzy systems with time-varying delays and redundant channels was studied in [9]. In [10], the focus was on the distributed robust fusion estimation problem that involves both stochastic and deterministic parameter uncertainties.

It is well known that in sensor networks, each node not only can obtain its own information in a timely manner, but also can acquire the information of adjacent nodes based on the topological structure. However, most of the research on sensor networks is based on a fixed topological structure. In practical engineering applications, the topological structure of sensor networks is not fixed but is in a dynamic state. For example, a sensor node may fail, the communication links between nodes may fluctuate, and new nodes may be added according to the requirements. However, due to the complexity of model construction and computation, the research on topological switching phenomena is relatively scarce; see, e.g., refs. [13,14,15,16]. This observation motivates us to focus on the present topic. It is worth emphasizing that a sensor network typically consists of numerous sensing nodes distributed around the target plant. As a result, the volume of measurement data acquired from these sensors can be extremely large.

Given the constrained bandwidth of the communication network, such heavy data transmission demands may give rise to packet losses, nonlinear distortions, and channel fading, all of which can degrade the filtering or estimation performance, as noted earlier. A considerable amount of research has been dedicated to this topic; representative works include [17,18,19,20,21,22,23,24,25]. For instance, an event-triggered distributed state estimation approach for general linear systems with deception attacks and partial measurements was proposed in [7]. In [18], the focus was on the resilient event-triggered distributed state estimation problem for nonlinear systems subjected to denial-of-service (DoS) attacks, whereas [19] addressed the distributed $H_{\infty }$-consensus state estimation problem for discrete time-varying systems with integral measurements over sensor networks. However, although event-triggering strategies can alleviate such problems to some degree, fixed thresholds still tend to cause unnecessary consumption of communication resources. Consequently, adaptively tuning the triggering threshold in accordance with system demands has emerged as a new challenge. In [20], the authors investigated the distributed adaptive event-triggered $H_{\infty }$ filtering problem for sector-bounded nonlinear systems over a filtering network with time-varying and switching topologies. The design of a secure adaptive event-triggered filter subject to input constraints and hybrid cyber attacks was presented in [21]. Additionally, an adaptive event-triggered scheme targeting the limited network bandwidth issue was introduced in [23].

In the practical deployment of modern sensor networks, particularly within complex industrial cyber–physical systems, data transmission between nodes frequently occurs over open and shared wireless communication channels. This inherent exposure leaves the networked architecture highly susceptible to malicious interference and sophisticated cyber-attacks, which can severely compromise data integrity and system stability. Among various cyber threats, the replay attack is particularly insidious because the adversary does not require prior knowledge of the target plant’s exact mathematical model; instead, they stealthily intercept, record, and reinject legitimate historical data packets to deceive the estimator and disrupt the state tracking process. To address this critical vulnerability, significant research efforts have been mobilized to design fault-tolerant and secure estimation frameworks; see, e.g., refs. [26,27]. In [26], the replay attack model was established, solving the problem of distributed Kalman fusion estimation in CPS under bandwidth limitations and replay attacks. In [27], the problem of distributed security state estimation for information network physical systems under replay attacks has been studied.

On another active research front, nonlinearities are often unavoidable in practical engineering systems. Consequently, it becomes crucial to examine the adverse effects that such nonlinearities may impose on system performance; representative studies can be found in [28,29,30,31,32,33,34]. For example, the robust $H_{\infty }$ finite-horizon output feedback control problem for a class of uncertain discrete stochastic nonlinear time-varying systems with both sensor and actuator saturations was investigated in [28]. Similarly, the finite-horizon $H_{\infty }$ fault estimation problem for nonlinear stochastic time-varying systems subject to randomly occurring faults and fading channels was studied in [29]. In spite of these contributions, current techniques are not fully adequate when extended to the adaptive event-triggered distributed estimation problem for discrete time-varying systems over sensor networks suffering from replay attacks. To fill this research void, the present work proposes a novel estimation framework.

To summarize, this paper addresses the design of an adaptive event-triggered distributed estimator for discrete time-varying systems deployed over sensor networks with randomly switching topologies and replay attacks, relying exclusively on the measurement outputs of sensor nodes. *The main contributions of this work are as follows: (1) a distributed estimation framework is constructed for sensor networks facing replay attacks; (2) an adaptive event-triggered mechanism is incorporated into the estimator design, which effectively cuts down the communication bandwidth needed by the network; (3) sufficient conditions are established to ensure that the estimation error dynamics satisfy the prescribed average $H_{\infty }$ performance.*

The structure of the remainder of this paper is as follows. In Section 2, the discrete time-varying system over sensor networks is described and the adaptive event-triggered distributed estimation problem is formulated. The main results, obtained via recursive linear matrix inequalities (RLMIs), are reported in Section 3. Section 4 provides a simulation example to validate the estimator design, and Section 5 summarizes the paper.
**Notation:**$R^{a},R^{a\times b}$The *a*-dim Euclidean space
and a×b real matrices${∥\alpha ∥}_{2}$${\left(\sum_{\zeta =0}^{N}{∥\alpha ∥}^{2}\right)}^{\frac{1}{2}}$ for $\alpha ={\left\{\alpha_{\zeta }\right\}}_{0\leq \zeta \leq N}$W≥V(W>V)W−V is positive semi-definite
(positive definite)T,I,0Transpose, identity, zero matrixE{x}Expectation⊗Kronecker product∗Symmetric entries in block matrices$l_{2}\left[0,N\right)$Space of square-summable sequences$1_{n}$${\left[1,\ldots ,1\right]}^{T}\in R^{n}$Prob{·}Probability of the event “·”

## 2. Problem Formulation

In this work, the sensor network is composed of *n* spatially deployed sensor nodes, whose communication structure alternates randomly between two directed graph topologies, denoted by $G^{s}=\left(V^{s},E^{s},A^{s}\right)$ for s=1,2. Here, *s* indexes the active topology. For both topologies, the node set is $V^{s}=\left\{1,2,\ldots ,n\right\}$ with s=1,2, the edge set satisfies $E^{s}\subseteq V^{s}\times V^{s}$, and the weighted adjacency matrix is given by $A^{s}=\left[a_{ij}^{s}\right]$, where all adjacency elements $a_{ij}^{s}$ are nonnegative. An edge of $G^{s}$ is represented by an ordered pair (i,j). The corresponding adjacency weight is strictly positive, i.e., $a_{ij}^{s}>0⟺\left(i,j\right)\in E^{s}$ for s=1,2, indicating that under the *s*th topology, sensor *i* can obtain data from sensor *j*. Moreover, it is assumed that $a_{ii}^{s}=1$ for every $i\in V^{s}$ and s=1,2, meaning that each node is considered to have a self-loop. The neighborhood of node $i\in V^{s}$, which includes the node itself, is defined as $N_{i}^{s}=\left\{j\in V^{s}:\left(i,j\right)\in E^{s}\right\}$.

Let a finite time-horizon be represented by [0,N]:={0,1,2,⋯,N}. The target plant under consideration is modeled by the following discrete time-varying stochastic system over k∈[0,N]:

(1) $$\left\{\begin{array}{cc} x_{\zeta +1} & =A_{\zeta }x_{\zeta }+B_{\zeta }f_{x_{\zeta }}+C_{\zeta }w_{\zeta }\\ z_{\zeta } & =M_{\zeta }x_{\zeta } \end{array}\right.$$

where $x_{\zeta }\in R^{n_{x}}$ denotes the state vector, which is not directly measurable; $z_{\zeta }\in R^{n_{z}}$ stands for the output to be estimated; $w_{\zeta }\in R^{n_{w}}$ represents the noise signal belonging to $l_{2}\left[0,N-1\right]$. The nonlinear vector-valued function $f_{•}$ satisfies $f_{0}=0$ and obeys the following sector-bounded constraint:

(2) $${\left[f_{x}-f_{y}-N_{1,x-y}\right]}^{T}\left[f_{x}-f_{y}-N_{2,x-y}\right]\leq 0,\;\;\;\forall x,y\in R^{n}$$

In this paper, the model of sensor node *i* is given as follows:

(3) $$y_{i,\zeta }=D_{i,\zeta }x_{\zeta }+E_{i,\zeta }v_{\zeta }$$

where $y_{i,\zeta }\in R^{n_{y}}$ is the output measured by sensor *i*, and $v_{\zeta }\in l_{2}\left[0,N-1\right]$ is an external disturbance. Moreover, all the mentioned matrices ($A_{\zeta }$, $B_{\zeta }$, $C_{\zeta }$, $M_{\zeta }$, $D_{i,\zeta }$ and $E_{i,\zeta }$) are known, real and time-varying with appropriate dimensions. The stochastic variable $\alpha_{\zeta }$ takes values of 0 or 1 with

(4) $$\mathrm{Prob}\left\{\alpha_{\zeta }=1\right\}=\bar{\alpha },\;\mathrm{Prob}\left\{\alpha_{\zeta }=0\right\}=1-\bar{\alpha }$$

where $\bar{\alpha }\left(0<\bar{\alpha }<1\right)$ is a known non-negative constant.

In order to reduce the communication rate, the following adaptive event generator function $g_{i}\left(\cdot ,\cdot \right)\;\left(i=1,2,\ldots ,n\right)$ is constructed:

(5) $$g_{i}\left(Y_{i,\zeta },\vartheta_{i}\right)=Y_{i,\zeta }^{T}\Omega_{i,\zeta }Y_{i,\zeta }-\vartheta_{i}r_{i,\zeta }^{T}\Omega_{i,\zeta }r_{i,\zeta }$$

where

(6) $$\begin{array}{cc} r_{i,\zeta }= & y_{i,\zeta }-D_{i,\zeta }{\hat{x}}_{i,\zeta }\\ Y_{i,\zeta }= & r_{i,\zeta_{u}^{i}}-r_{i,\zeta } \end{array}$$

The innovation sequence transmitted over the network is denoted by $r_{i,\zeta }$, while ${\hat{x}}_{i,\zeta }\in R^{n_{x}}$ represents the state estimate computed at sensor node *i*. Furthermore, $r_{i,\zeta_{u}^{j}}$ corresponds to the most recently broadcast innovation at the current event instant. The matrices $\Omega_{i,\zeta }$(i=1,2,…,n) are symmetric and positive-definite, and $\vartheta_{i}$ serves as the adaptive event-triggered threshold that determines the triggering rate, satisfying

(7) $$\vartheta_{i,\zeta +1}=\vartheta_{i,\zeta }+h_{i,\zeta }$$

where $\vartheta_{i,0}\in \left[0,1\right]$ is the initial value of the trigger parameter and

(8) $$h_{i,\zeta }=\left\{\begin{array}{cc} \varepsilon , & Y_{i,\zeta }^{T}Y_{i,\zeta }<\rho \\ 0, & Y_{i,\zeta }^{T}Y_{i,\zeta }=\rho \\ -\varepsilon , & Y_{i,\zeta }^{T}Y_{i,\zeta }>\rho \end{array}\right.$$

where ρ and ε are non-negative constants. The execution is triggered whenever the following condition holds:

(9) $$g_{i}\left(Y_{i,\zeta },\vartheta_{i}\right)>0$$

Consequently, the sequence of adaptive event-triggered instants $0\leq \zeta_{0}^{i}\leq \zeta_{1}^{i}\leq \cdots \leq \zeta_{\zeta }^{i}\leq \cdots$ can be obtained through the following iterative rule:

(10) $$\zeta_{\zeta +1}^{i}=\min\left\{\zeta \in N^{0}|\zeta >\zeta_{\zeta }^{i},g_{i}\left(Y_{i,\zeta },\vartheta_{i}\right)>0\right\}$$

In non-ideal network environments, sensor measurement data $r_{i,\zeta_{\zeta }^{i}}$ may be threatened by replay attacks, leading to estimator errors. The input cannot obtain reliable data. When a replay attack occurs between the sensor and the estimator at time ζ, the attack behavior is described as follows:

- Clear the network transmission packets at the current time ζ.
- Select a data packet earlier in time from the stored historical data packets $Q_{\zeta }$ and send it to the estimator.
- Update stored historical data packets $Q_{\zeta +1}=\left(Q_{\zeta }\setminus r_{i,\zeta_{\varepsilon }^{i}}\right)\cup r_{i,\zeta_{\zeta }^{i}}$, where $\zeta_{\varepsilon }^{i}=\min\left\{\zeta_{1}^{i},\zeta_{2}^{i},\ldots ,\zeta_{l}^{i}\right\}$. Due to limited storage memory, assume the attacker can only store *l* historical data packets. The historical data packet stores a set of data represented as $Q_{\zeta }=\left\{r_{i,\zeta_{1}^{i}},r_{i,\zeta_{2}^{i}},\ldots ,r_{i,\zeta_{l}^{i}}\right\}$. To effectively reduce the impact of replay attacks on estimation performance, the following compensation terms are constructed:

(11) $${\underline{r}}_{i,\zeta }^{c}={\underline{r}}_{i,\zeta -1}$$

The measurement received by the estimator is

(12) $$\begin{array}{c} {\underline{r}}_{i,\zeta }=\alpha_{\zeta }{\underline{r}}_{i,\zeta }^{c}+\left(1-\alpha_{\zeta }\right)r_{i,\zeta_{\zeta }^{i}} \end{array}$$

In this work, the estimator implemented at sensor node *i* takes the following form:

(13) $$\left\{\begin{array}{cc} {\hat{x}}_{i,\zeta +1} & =A_{\zeta }{\hat{x}}_{i,\zeta }+\beta_{\zeta }\left(\sum_{j\in N_{i}^{1}}a_{ij}^{1}K_{ij,\zeta }^{1}{\underline{r}}_{j,\zeta }\right)+\left(1-\beta_{\zeta }\right)\left(\sum_{j\in N_{i}^{2}}a_{ij}^{2}K_{ij,\zeta }^{2}{\underline{r}}_{j,\zeta }\right)+B_{\zeta }f_{{\hat{x}}_{i,\zeta }}\\ {\hat{z}}_{i,\zeta } & =M_{\zeta }{\hat{x}}_{i,\zeta } \end{array}\right.$$

where ${\hat{x}}_{i,\zeta }\in R^{n_{x}}$ and ${\hat{z}}_{i,\zeta }\in R^{n_{z}}$ correspond to the state estimate and the estimate of $z_{\zeta }$ at sensor node *i*, respectively. $K_{ij,\zeta }^{s_{\zeta }}$$\left(s_{\zeta }=1,2\right)$ denote the estimator gains to be designed for node *i*. To describe the phenomenon of switching topologies, a Bernoulli-distributed stochastic variable $\beta_{\zeta }\in R$ is employed, whose probability distribution is given by

(14) $$\mathrm{Prob}\left\{\beta_{\zeta }=1\right\}=\bar{\beta },\;\mathrm{Prob}\left\{\beta_{\zeta }=0\right\}=1-\bar{\beta }$$

where $\bar{\beta }\left(0<\bar{\beta }<1\right)$ is a known constant which can be obtained by statistical experiments.

Letting $e_{i,\zeta }=x_{\zeta }-{\hat{x}}_{i,\zeta }$, $f_{e_{i,\zeta }}=f_{x_{\zeta }}-f_{{\hat{x}}_{i,\zeta }}$, we have

(15) $$\left\{\begin{array}{cc} e_{i,\zeta +1} & =A_{\zeta }e_{i,\zeta }+B_{\zeta }f_{e_{i,\zeta }}+C_{\zeta }w_{\zeta }-\beta_{\zeta }\left(\sum_{j\in N_{i}^{1}}a_{ij}^{1}K_{ij,\zeta }^{1}{\underline{r}}_{j,\zeta }\right)\\ \; & -\left(1-\beta_{\zeta }\right)\left(\sum_{j\in N_{i}^{2}}a_{ij}^{2}K_{ij,\zeta }^{2}{\underline{r}}_{j,\zeta }\right)\\ {\tilde{z}}_{i,\zeta } & =M_{\zeta }e_{i,\zeta } \end{array}\right.$$

For convenience of later analysis, denote

(16) $$\begin{array}{cc} \; & {\hat{x}}_{\zeta }={\left[\begin{array}{cccc} {\hat{x}}_{1,\zeta }^{T} & {\hat{x}}_{2,\zeta }^{T} & \cdots & {\hat{x}}_{n,\zeta }^{T} \end{array}\right]}^{T},\;{\bar{A}}_{\zeta }=I_{n}⊗A_{\zeta },\;{\bar{B}}_{\zeta }=I_{n}⊗B_{\zeta },\\ \; & {\hat{z}}_{\zeta }={\left[\begin{array}{cccc} {\hat{z}}_{1,\zeta }^{T} & {\hat{z}}_{2,\zeta }^{T} & \cdots & {\hat{z}}_{n,\zeta }^{T} \end{array}\right]}^{T},\;{\bar{C}}_{\zeta }=I_{n}⊗C_{\zeta },\;{\bar{M}}_{\zeta }=I_{n}⊗M_{\zeta },\\ \; & {\bar{\underline{r}}}_{\zeta }={\left[\begin{array}{cccc} {\underline{r}}_{1,\zeta }^{T} & {\underline{r}}_{2,\zeta }^{T} & \cdots & {\underline{r}}_{n,\zeta }^{T} \end{array}\right]}^{T},\;{\bar{w}}_{\zeta }=1_{n}⊗w_{\zeta },\;{\bar{v}}_{\zeta }=1_{n}⊗v_{\zeta },\\ \; & e_{\zeta }={\left[\begin{array}{cccc} e_{1,\zeta }^{T} & e_{2,\zeta }^{T} & \cdots & e_{n,\zeta }^{T} \end{array}\right]}^{T},\;{\bar{x}}_{\zeta }=1_{n}⊗x_{\zeta },\;{\bar{z}}_{\zeta }=1_{n}⊗z_{\zeta },\\ \; & {\bar{Y}}_{\zeta }={\left[\begin{array}{cccc} Y_{1,\zeta }^{T} & Y_{2,\zeta }^{T} & \cdots & Y_{n,\zeta }^{T} \end{array}\right]}^{T},{\bar{D}}_{\zeta }=\mathrm{diag}\left\{D_{1,\zeta },D_{2,\zeta },\ldots ,D_{n,\zeta }\right\},\\ \; & f_{e_{\zeta }}={\left[\begin{array}{cccc} f_{e_{1,\zeta }}^{T} & f_{e_{2,\zeta }}^{T} & \cdots & f_{e_{n,\zeta }}^{T} \end{array}\right]}^{T},\;{\bar{N}}_{1}=I_{n}⊗N_{1},\\ \; & {\bar{E}}_{\zeta }=\mathrm{diag}\left\{E_{1,\zeta },E_{2,\zeta },\ldots ,E_{n,\zeta }\right\},\;{\bar{N}}_{2}=I_{n}⊗N_{2},\\ \; & {\bar{K}}_{\zeta }^{1}={\left[{\bar{K}}_{ij,\zeta }^{1}\right]}_{n_{x}\times n_{y}}\;\mathrm{with}\;{\bar{K}}_{ij,\zeta }^{1}=\{\begin{array}{c} a_{ij}^{1}K_{ij,\zeta }^{1},\;\;i=1,2,\ldots ,n;\;\;j\in N_{i}^{1}\\ 0,\;\;\;\;\;\;\;\;\;\;i=1,2,\ldots ,n;\;\;j\notin N_{i}^{1} \end{array}\\ \; & {\bar{K}}_{\zeta }^{2}={\left[{\bar{K}}_{ij,\zeta }^{2}\right]}_{n_{x}\times n_{y}}\;\mathrm{with}\;{\bar{K}}_{ij,\zeta }^{2}=\{\begin{array}{c} a_{ij}^{2}K_{ij,\zeta }^{2},\;\;i=1,2,\ldots ,n;\;\;j\in N_{i}^{2}\\ 0,\;\;\;\;\;\;\;\;\;\;i=1,2,\ldots ,n;\;\;j\notin N_{i}^{2}. \end{array} \end{array}$$

Obviously, since $a_{ij}^{1}=a_{ij}^{2}=0$ when $j\notin N_{i}^{1},N_{i}^{2}$ respectively, ${\bar{K}}_{\zeta }^{1}$ and ${\bar{K}}_{\zeta }^{2}$ can be expressed as

(17) $${\bar{K}}_{\zeta }^{1}\in T_{n_{x}\times n_{y}},\;{\bar{K}}_{\zeta }^{2}\in T_{n_{x}\times n_{y}}$$

where $T_{p\times q}=\left\{\bar{T}=\left[T_{ij}\right]\in R^{np\times nq}|T_{ij}\in R^{p\times q},\;T_{ij}=0\;\;\mathrm{if}\;j\notin N_{i}\right\}$.

Letting $e_{\zeta }={\bar{x}}_{\zeta }-{\hat{x}}_{\zeta }$, ${\tilde{z}}_{\zeta }={\bar{z}}_{\zeta }-{\hat{z}}_{\zeta }$, we have

(18) $$\left\{\begin{array}{cc} e_{\zeta +1}= & {\bar{A}}_{\zeta }e_{\zeta }+{\bar{C}}_{\zeta }{\bar{w}}_{\zeta }-\left(\beta_{\zeta }{\bar{K}}_{\zeta }^{1}+\left(1-\beta_{\zeta }\right){\bar{K}}_{\zeta }^{2}\right)\left(1-\alpha_{\zeta }\right){\bar{D}}_{\zeta }e_{\zeta }\\ \; & -\left(\beta_{\zeta }{\bar{K}}_{\zeta }^{1}+\left(1-\beta_{\zeta }\right){\bar{K}}_{\zeta }^{2}\right)\alpha_{\zeta }{\bar{\underline{r}}}_{\zeta -1}-\left(\beta_{\zeta }{\bar{K}}_{\zeta }^{1}+\left(1-\beta_{\zeta }\right){\bar{K}}_{\zeta }^{2}\right)\\ \; & \times \left(1-\alpha_{\zeta }\right){\bar{E}}_{\zeta }{\bar{v}}_{\zeta }-\left(\beta_{\zeta }{\bar{K}}_{\zeta }^{1}+\left(1-\beta_{\zeta }\right){\bar{K}}_{\zeta }^{2}\right)\left(1-\alpha_{\zeta }\right){\bar{Y}}_{\zeta }\\ \; & +{\bar{B}}_{\zeta }f_{e_{\zeta }}\\ {\tilde{z}}_{\zeta }= & {\bar{M}}_{\zeta }e_{\zeta } \end{array}\right.$$

By defining $\eta_{\zeta }={\left[e_{\zeta }^{T}\;\;{\bar{\underline{r}}}_{\zeta -1}^{T}\right]}^{T}$, the estimation error dynamics of the entire sensor network can be described by the following augmented system:

(19) $$\left\{\begin{array}{cc} \eta_{\zeta +1} & =\left(A_{1,\zeta }+A_{2,\zeta }^{*}\right)\eta_{\zeta }+\left(B_{1,\zeta }+B_{2,\zeta }^{*}\right)w_{v,\zeta }+\left(C_{1,\zeta }+C_{2,\zeta }^{*}\right){\bar{Y}}_{\zeta }+I_{1}{\bar{B}}_{\zeta }{\bar{f}}_{I_{1}^{T}\eta_{\zeta }}\\ {\tilde{z}}_{\zeta } & =M_{\zeta }\eta_{\zeta } \end{array}\right.$$

where

(20) $$\begin{array}{cc} \; & A_{1,\zeta }=\left[\begin{array}{cc} {\bar{A}}_{\zeta }-{\bar{K}}_{\zeta ,\beta_{\zeta }}\left(1-\bar{\alpha }\right){\bar{D}}_{\zeta } & -{\bar{K}}_{\zeta ,\beta_{\zeta }}\bar{\alpha }\\ \left(1-\bar{\alpha }\right){\bar{D}}_{\zeta } & \bar{\alpha }I_{n} \end{array}\right],\;C_{1,\zeta }=\left[\begin{array}{c} -{\bar{K}}_{\zeta ,\beta_{\zeta }}\left(1-\bar{\alpha }\right)\\ \left(1-\bar{\alpha }\right)I_{n} \end{array}\right]\\ \; & B_{1,\zeta }=\left[\begin{array}{cc} {\bar{C}}_{\zeta } & -{\bar{K}}_{\zeta ,\beta_{\zeta }}\left(1-\bar{\alpha }\right){\bar{E}}_{\zeta }\\ 0 & \left(1-\bar{\alpha }\right){\bar{E}}_{\zeta } \end{array}\right],\;C_{2,\zeta }^{*}=\left[\begin{array}{c} {\bar{K}}_{\zeta ,\beta_{\zeta }}\left(\alpha_{\zeta }-\bar{\alpha }\right)\\ \left(\bar{\alpha }-\alpha_{\zeta }\right)I_{n} \end{array}\right],\\ \; & A_{2,\zeta }^{*}=\left[\begin{array}{cc} {\bar{K}}_{\zeta ,\beta_{\zeta }}\left(\alpha_{\zeta }-\bar{\alpha }\right){\bar{D}}_{\zeta } & {\bar{K}}_{\zeta ,\beta_{\zeta }}\left(\bar{\alpha }-\alpha_{\zeta }\right)\\ \left(\bar{\alpha }-\alpha_{\zeta }\right){\bar{D}}_{\zeta } & \left(\alpha_{\zeta }-\bar{\alpha }\right)I_{n} \end{array}\right],\;M_{\zeta }=\left[\begin{array}{cc} {\bar{M}}_{\zeta } & 0 \end{array}\right],\\ \; & B_{2,\zeta }^{*}=\left[\begin{array}{cc} 0 & {\bar{K}}_{\zeta ,\beta_{\zeta }}\left(\alpha_{\zeta }-\bar{\alpha }\right){\bar{E}}_{\zeta }\\ 0 & \left(\bar{\alpha }-\alpha_{\zeta }\right){\bar{E}}_{\zeta } \end{array}\right],\;I_{1}=\left[\begin{array}{c} I\\ 0 \end{array}\right],\;w_{v,\zeta }=\left[\begin{array}{c} {\bar{w}}_{\zeta }\\ {\bar{v}}_{\zeta } \end{array}\right],\\ \; & {\bar{f}}_{I_{1}^{T}\eta_{\zeta }}=f_{e_{\zeta }}={\left[\begin{array}{cccc} f_{e_{1,\zeta }}^{T} & f_{e_{2,\zeta }}^{T} & \cdots & f_{e_{n,\zeta }}^{T} \end{array}\right]}^{T},\\ \; & {\bar{K}}_{\zeta ,\beta_{\zeta }}=\beta_{\zeta }{\bar{K}}_{\zeta }^{1}+\left(1-\beta_{\zeta }\right){\bar{K}}_{\zeta }^{2}. \end{array}$$

Prior to the subsequent analysis, we first present the following definition. Moreover, from (2) one obtains

(21) $${\left[{\bar{f}}_{I_{1}^{T}\eta_{\zeta }}-{\bar{N}}_{1}I_{1}^{T}\eta_{\zeta }\right]}^{T}\left[{\bar{f}}_{I_{1}^{T}\eta_{\zeta }}-{\bar{N}}_{2}I_{1}^{T}\eta_{\zeta }\right]<0$$

**Definition** **1.** *Given a disturbance attenuation level γ>0 and a set of positive definite matrices $S_{i}>0$ (0≤i≤n), the estimation error ${\tilde{z}}_{i,\zeta }=z_{\zeta }-{\hat{z}}_{i,\zeta }$ is said to fulfill the average $H_{\infty }$ performance requirements if the following condition is met:*

(22) $$\frac{1}{n}\sum_{i=1}^{n}E\{∥{\tilde{z}}_{i,\zeta }{∥}_{\left[0,N-1\right]}^{2}\}<\gamma^{2}\left\{∥w_{v,\zeta }{∥}_{\left[0,N-1\right]}^{2}+\frac{1}{n}\sum_{i=1}^{n}{\left(x_{0}-{\hat{x}}_{i,0}\right)}^{T}S_{i}\left(x_{0}-{\hat{x}}_{i,0}\right)\right\}$$

The average $H_{\infty }$ performance requirement (22) can be expressed equivalently as

(23) $$J:=E\{∥{\tilde{z}}_{\zeta }{∥}_{\left[0,N-1\right]}^{2}\}-\gamma^{2}\{∥w_{v,\zeta }{∥}_{\left[0,N-1\right]}^{2}+e_{0}^{T}Re_{0}\}<0$$

where $e_{0}={\bar{x}}_{0}-{\hat{x}}_{0}$ and $R=\mathrm{diag}\{S_{1},S_{2},\ldots ,S_{n}\}$.

This paper aims to determine the estimator gains $K_{ij,\zeta }^{1}$ and $K_{ij,\zeta }^{2}$ such that the estimation error dynamics (19) meet the performance specifications (23).

## 3. Main Results

The main results are built upon the following lemmas.

**Lemma** **1** (Schur Complement)**.** *Let $S_{1},S_{2},S_{3}$ be constant matrices with $S_{1}=S_{1}^{T}$ and $0<S_{2}=S_{2}^{T}$. Then the inequality $S_{1}+S_{3}^{T}S_{2}^{-1}S_{3}<0$ holds if and only if*

(24) $$\left[\begin{array}{cc} S_{1} & S_{3}^{T}\\ S_{3} & -S_{2} \end{array}\right]<0\;\mathrm{or}\;\left[\begin{array}{cc} -S_{2} & S_{3}\\ S_{3}^{T} & S_{1} \end{array}\right]<0.$$

**Lemma** **2.** *Let $P=diag\{P_{1},P_{2},\ldots ,P_{n}\}$ where each $P_{i}\in R^{p\times p}$(1≤i≤n) is invertible. If X=PW with $W\in R^{np\times nq}$, then $W\in T_{p\times q}⟺X\in T_{p\times q}$.*

Before proceeding, we denote

$$\begin{array}{cc} & r_{\zeta }={\left[\begin{array}{cccc} r_{1,\zeta }^{T} & r_{2,\zeta }^{T} & \cdots & r_{n,\zeta }^{T} \end{array}\right]}^{T},\;\Omega_{\zeta }=\mathrm{diag}\left\{\Omega_{1,\zeta },\Omega_{2,\zeta },\ldots ,\Omega_{n,\zeta }\right\},\\ & \bar{\vartheta }=\mathrm{diag}\left\{\vartheta_{1}I,\vartheta_{2}I,\ldots ,\vartheta_{n}I\right\}. \end{array}$$

The theorem presented below provides a sufficient condition for the augmented system (19) to fulfill the average $H_{\infty }$ performance requirements specified in Definition 1.

**Theorem** **1.** *For the discrete time-varying stochastic system (1) and sensor measurement model (3) with event-triggering mechanism (5), assume the estimator gains $K_{ij,\zeta }^{s_{\zeta }}$$\left(s_{\zeta }=1,2\right)$ are given. Then the augmented estimation error dynamics (19) meet the average $H_{\infty }$ performance requirements (22) with initial condition $\eta_{0}^{T}P_{0}\eta_{0}\leq \gamma^{2}e_{0}^{T}Re_{\zeta }$, if there exist a set of positive definite matrices ${\left\{P_{\zeta }\right\}}_{0\leq \zeta \leq N+1}$ together with scalars ϵ>0, λ>0 such that*

(25) $$\Theta_{\zeta }=\left[\begin{array}{cccc} \Theta_{11,\zeta } & * & * & *\\ \Theta_{21,\zeta } & \Theta_{22,\zeta } & * & *\\ \Theta_{31,\zeta } & \Theta_{32,\zeta } & \Theta_{33,\zeta } & *\\ \Theta_{41,\zeta } & \Theta_{42,\zeta } & \Theta_{43,\zeta } & \Theta_{44,\zeta } \end{array}\right]<0,\;\;\mathrm{for}\;0\leq \zeta \leq N$$

*where*

$$\begin{array}{cc} \Theta_{11,\zeta } & =A_{1,\zeta }^{T}P_{\zeta +1}A_{1,\zeta }+A_{2,\zeta }^{T}P_{\zeta +1}A_{2,\zeta }-P_{\zeta }\\ & +\epsilon D_{\zeta }^{T}\bar{\vartheta }\Omega_{\zeta }D_{\zeta }-\frac{1}{2}\lambda I_{1}\left({\bar{N}}_{1}^{T}{\bar{N}}_{2}+{\bar{N}}_{2}^{T}{\bar{N}}_{1}\right)I_{1}^{T}+M_{\zeta }^{T}M_{\zeta },\;\\ \Theta_{21,\zeta } & =B_{1,\zeta }^{T}P_{\zeta +1}A_{1,\zeta }+B_{2,\zeta }^{T}P_{\zeta +1}A_{2,\zeta }+\epsilon E_{\zeta }^{T}\bar{\vartheta }\Omega_{\zeta }D_{\zeta },\;\\ \Theta_{22,\zeta } & =B_{1,\zeta }^{T}P_{\zeta +1}B_{1,\zeta }+B_{2,\zeta }^{T}P_{\zeta +1}B_{2,\zeta }+\epsilon E_{\zeta }^{T}\bar{\vartheta }\Omega_{\zeta }E_{\zeta }-\gamma^{2}I,\\ \Theta_{31,\zeta } & =C_{1,\zeta }^{T}P_{\zeta +1}A_{1,\zeta }+C_{2,\zeta }^{T}P_{\zeta +1}A_{2,\zeta },\;\\ \Theta_{32,\zeta } & =C_{1,\zeta }^{T}P_{\zeta +1}B_{1,\zeta }+C_{2,\zeta }^{T}P_{\zeta +1}B_{2,\zeta },\Theta_{33,\zeta }=C_{1,\zeta }^{T}P_{\zeta +1}C_{1,\zeta }+C_{2,\zeta }^{T}P_{\zeta +1}C_{2,\zeta }-\epsilon \Omega_{\zeta },\;\\ \Theta_{41,\zeta } & ={\left(I_{1}{\bar{B}}_{\zeta }\right)}^{T}P_{\zeta +1}A_{1,\zeta }+\frac{1}{2}\lambda \left({\bar{N}}_{1}+{\bar{N}}_{2}\right)I_{1}^{T},\Theta_{42,\zeta }={\left(I_{1}{\bar{B}}_{\zeta }\right)}^{T}P_{\zeta +1}B_{1,\zeta },\;\\ \Theta_{43,\zeta } & ={\left(I_{1}{\bar{B}}_{\zeta }\right)}^{T}P_{\zeta +1}C_{1,\zeta },\Theta_{44,\zeta }={\left(I_{1}{\bar{B}}_{\zeta }\right)}^{T}P_{\zeta +1}\left(I_{1}{\bar{B}}_{\zeta }\right)-\lambda I,\;\\ A_{2,\zeta } & =\left[\begin{array}{cc} {\tilde{\alpha }}_{\zeta }{\bar{K}}_{\zeta }\left(\beta_{\zeta }\right){\bar{D}}_{\zeta } & -{\tilde{\alpha }}_{\zeta }{\bar{K}}_{\zeta }\left(\beta_{\zeta }\right)\\ -{\tilde{\alpha }}_{\zeta }{\bar{D}}_{\zeta } & {\tilde{\alpha }}_{\zeta }I_{n} \end{array}\right],\;C_{2,\zeta }=\left[\begin{array}{c} {\tilde{\alpha }}_{\zeta }{\bar{K}}_{\zeta }\left(\beta_{\zeta }\right)\\ -{\tilde{\alpha }}_{\zeta }I_{n} \end{array}\right],\;\\ B_{2,\zeta } & =\left[\begin{array}{cc} 0 & {\tilde{\alpha }}_{\zeta }{\bar{K}}_{\zeta }\left(\beta_{\zeta }\right){\bar{E}}_{\zeta }\\ 0 & -{\tilde{\alpha }}_{\zeta }{\bar{E}}_{\zeta } \end{array}\right],{\tilde{\alpha }}_{\zeta }=\alpha_{\zeta }-\bar{\alpha }.\; \end{array}$$

**Proof.**  Define

(26) $$J_{\zeta }=\eta_{\zeta +1}^{T}P_{\zeta +1}\eta_{\zeta +1}-\eta_{\zeta }^{T}P_{\zeta }\eta_{\zeta }$$

Taking the mathematical expectation of $J_{\zeta }$ along the trajectories of the augmented system (19) yields

(27) $$\begin{array}{cc} E\{J_{\zeta }\}= & E\{\eta_{\zeta +1}^{T}P_{\zeta +1}\eta_{\zeta +1}-\eta_{\zeta }^{T}P_{\zeta }\eta_{\zeta }\}\\ = & E\{(\left(A_{1,\zeta }+A_{2,\zeta }^{*}\right)\eta_{\zeta }+\left(B_{1,\zeta }+B_{2,\zeta }^{*}\right)w_{v,\zeta }+(C_{1,\zeta }\\ \; & +C_{2,\zeta }^{*}){\bar{Y}}_{\zeta }+I_{1}{\bar{B}}_{\zeta }{\bar{f}}_{I_{1}^{T}\eta_{\zeta }}{)}^{T}P_{\zeta +1}(\left(A_{1,\zeta }+A_{2,\zeta }^{*}\right)\eta_{\zeta }\\ \; & +\left(B_{1,\zeta }+B_{2,\zeta }^{*}\right)w_{v,\zeta }+\left(C_{1,\zeta }+C_{2,\zeta }^{*}\right){\bar{Y}}_{\zeta }+I_{1}{\bar{B}}_{\zeta }{\bar{f}}_{I_{1}^{T}\eta_{\zeta }})\\ \; & -\eta_{\zeta }^{T}P_{\zeta }\eta_{\zeta }\} \end{array}$$

From the adaptive event-triggering condition (5), we have

(28) $${\bar{Y}}_{\zeta }^{T}\Omega_{\zeta }{\bar{Y}}_{\zeta }-r_{\zeta }^{T}\bar{\vartheta }\Omega_{\zeta }r_{\zeta }<0$$

and hence

(29) $$r_{\zeta }=D_{\zeta }\eta_{\zeta }+E_{\zeta }w_{v,\zeta }$$

where

(30) $$\begin{array}{cc} \; & D_{\zeta }=\left[\begin{array}{cc} {\bar{D}}_{\zeta } & 0 \end{array}\right],\;E_{\zeta }=\left[\begin{array}{cc} 0 & {\bar{E}}_{\zeta } \end{array}\right] \end{array}$$

Moreover, it can be derived that

(31) $$\begin{array}{cc} r_{\zeta }^{T}\bar{\vartheta }\Omega_{\zeta }r_{\zeta }= & {\left(D_{\zeta }\eta_{\zeta }+E_{\zeta }w_{v,\zeta }\right)}^{T}\bar{\vartheta }\Omega_{\zeta }\left(D_{\zeta }\eta_{\zeta }+E_{\zeta }w_{v,\zeta }\right) \end{array}$$

For a positive scalar ϵ and λ, combining (27), (28) and (31), it is easy to see that

(32) $$\begin{array}{cc} E\{J_{\zeta }\}<\; & E\left\{J_{\zeta }\right\}-\epsilon \left({\bar{Y}}_{\zeta }^{T}\Omega_{\zeta }{\bar{Y}}_{\zeta }-r_{\zeta }^{T}\bar{\vartheta }\Omega_{\zeta }r_{\zeta }\right)\\ \; & -\lambda {\left[{\bar{f}}_{I_{1}^{T}\eta_{\zeta }}-{\bar{N}}_{1}I_{1}^{T}\eta_{\zeta }\right]}^{T}\left[{\bar{f}}_{I_{1}^{T}\eta_{\zeta }}-{\bar{N}}_{2}I_{1}^{T}\eta_{\zeta }\right]\\ =\; & E\{(\left(A_{1,\zeta }+A_{2,\zeta }^{*}\right)\eta_{\zeta }+\left(B_{1,\zeta }+B_{2,\zeta }^{*}\right)w_{v,\zeta }+(C_{1,\zeta }\\ \; & +C_{2,\zeta }^{*}){\bar{Y}}_{\zeta }+I_{1}{\bar{B}}_{\zeta }{\bar{f}}_{I_{1}^{T}\eta_{\zeta }}{)}^{T}P_{\zeta +1}(\left(A_{1,\zeta }+A_{2,\zeta }^{*}\right)\eta_{\zeta }\\ \; & +\left(B_{1,\zeta }+B_{2,\zeta }^{*}\right)w_{v,\zeta }+\left(C_{1,\zeta }+C_{2,\zeta }^{*}\right){\bar{Y}}_{\zeta }+I_{1}{\bar{B}}_{\zeta }{\bar{f}}_{I_{1}^{T}\eta_{\zeta }})\\ \; & -\eta_{\zeta }^{T}P_{\zeta }\eta_{\zeta }-\epsilon {\bar{Y}}_{\zeta }^{T}\Omega_{\zeta }{\bar{Y}}_{\zeta }\\ \; & +\epsilon {\left(D_{\zeta }\eta_{\zeta }+E_{\zeta }w_{v,\zeta }\right)}^{T}\bar{\vartheta }\Omega_{\zeta }\left(D_{\zeta }\eta_{\zeta }+E_{\zeta }w_{v,\zeta }\right)\\ \; & -\lambda {\left[{\bar{f}}_{I_{1}^{T}\eta_{\zeta }}-{\bar{N}}_{1}I_{1}^{T}\eta_{\zeta }\right]}^{T}\left[{\bar{f}}_{I_{1}^{T}\eta_{\zeta }}-{\bar{N}}_{2}I_{1}^{T}\eta_{\zeta }\right]\} \end{array}$$

Adding the zero term ${\tilde{z}}_{\zeta }^{T}{\tilde{z}}_{\zeta }-\gamma^{2}w_{v,\zeta }^{T}w_{v,\zeta }-{\tilde{z}}_{\zeta }^{T}{\tilde{z}}_{\zeta }+\gamma^{2}w_{v,\zeta }^{T}w_{v,\zeta }\;\mathrm{to}\;E\left\{J_{\zeta }\right\}$ results in

(33) $$E\left\{J_{\zeta }\right\}<E\left\{{\bar{\eta }}_{\zeta }^{T}\Theta {\bar{\eta }}_{\zeta }-{\tilde{z}}_{\zeta }^{T}{\tilde{z}}_{\zeta }+\gamma^{2}w_{v,\zeta }^{T}w_{v,\zeta }\right\}$$

where

(34) $${\bar{\eta }}_{\zeta }={\left[\eta_{\zeta }^{T}\;w_{v,\zeta }^{T}\;{\bar{Y}}_{\zeta }^{T}\;{\bar{f}}_{I_{1}^{T}\eta_{\zeta }}^{T}\right]}^{T}$$

Summing both sides of (33) over ζ from 0 to N−1 leads to

(35) $$\begin{array}{cc} \sum_{\zeta =0}^{N-1}E\left\{J_{\zeta }\right\}= & E\{\eta_{N}^{T}P_{N}\eta_{N}-\eta_{0}^{T}P_{0}\eta_{0}\}\\ < & E\left\{\sum_{\zeta =0}^{N-1}{\bar{\eta }}_{\zeta }^{T}\Theta_{\zeta }{\bar{\eta }}_{\zeta }\right\}-E\left\{\sum_{\zeta =0}^{N-1}\left({\tilde{z}}_{\zeta }^{T}{\tilde{z}}_{\zeta }-\gamma^{2}w_{v,\zeta }^{T}w_{v,\zeta }\right)\right\} \end{array}$$

In light of the average $H_{\infty }$ performance constraints (23), one has

(36) $$J<E\left\{\sum_{\zeta =0}^{N-1}{\bar{\eta }}_{\zeta }^{T}\Theta_{\zeta }{\bar{\eta }}_{\zeta }\right\}-E\left\{\eta_{N}^{T}P_{N}\eta_{N}\right\}+\eta_{0}^{T}P_{0}\eta_{0}-\gamma^{2}e_{0}^{T}Re_{\zeta }$$

Since $P_{N}>0$ and the initial condition $\eta_{0}^{T}P_{0}\eta_{0}\leq \gamma^{2}e_{0}^{T}Re_{\zeta }$ is satisfied, it follows that J<0 whenever (25) holds. This completes the proof. □

Theorem 1 has established the performance analysis for the augmented estimation error system (19). Next, we proceed to determine the required estimator gains; the corresponding design result is stated in the following theorem.

**Theorem** **2.** *For the discrete time-varying stochastic system (1) and the sensor measurement model (3) combined with the adaptive event-triggering scheme (5), the estimation error dynamics (19) meet the average $H_{\infty }$ performance specifications (23) if there exist a family of positive definite matrices ${\left\{P_{\zeta }\right\}}_{0\leq \zeta \leq N+1}>0$, a matrix $L_{\zeta }\in T_{2nn_{x}\times n_{y}}$, and positive scalars ϵ>0, λ>0 such that the initial condition*

(37) $$\eta_{0}^{T}P_{0}\eta_{0}\leq \gamma^{2}e_{0}^{T}Re_{\zeta }$$

and the RLMIs

(38) $$\Xi_{\zeta }=\left[\begin{array}{cccccc} \Xi_{11,\zeta } & * & * & * & * & *\\ \Xi_{21,\zeta } & \Xi_{22,\zeta } & * & * & * & *\\ 0 & 0 & \Xi_{33,\zeta } & * & * & *\\ \Xi_{41,\zeta } & 0 & 0 & \Xi_{44,\zeta } & * & *\\ \Xi_{51,\zeta } & \Xi_{52,\zeta } & \Xi_{53,\zeta } & \Xi_{54,\zeta } & -P_{\zeta +1} & *\\ \Xi_{61,\zeta } & \Xi_{62,\zeta } & \Xi_{63,\zeta } & 0 & 0 & -P_{\zeta +1} \end{array}\right]<0$$

where

$$\begin{array}{cc} \Xi_{11,\zeta }= & -P_{\zeta }+\epsilon D_{\zeta }^{T}\bar{\vartheta }\Omega_{\zeta }D_{\zeta }-\frac{1}{2}\lambda I_{1}\left({\bar{N}}_{1}^{T}{\bar{N}}_{2}+{\bar{N}}_{2}^{T}{\bar{N}}_{1}\right)I_{1}^{T}+M_{\zeta }^{T}M_{\zeta },\;\\ \Xi_{21,\zeta }= & \epsilon E_{\zeta }^{T}\bar{\vartheta }\Omega_{\zeta }D_{\zeta },\;\Xi_{22,\zeta }=\epsilon E_{\zeta }^{T}\bar{\vartheta }\Omega_{\zeta }E_{\zeta }-\gamma^{2}I,\;\\ \Xi_{33,\zeta }= & -\epsilon \Omega_{\zeta },\;\Xi_{41,\zeta }=\frac{1}{2}\lambda \left({\bar{N}}_{1}+{\bar{N}}_{2}\right)I_{1}^{T},\;\Xi_{44,\zeta }=-\lambda I,\;\\ \Xi_{51,\zeta }= & P_{\zeta +1}A_{11,\zeta }+L_{\zeta }A_{12,\zeta },\;\Xi_{52,\zeta }=P_{\zeta +1}B_{11,\zeta }+L_{\zeta }B_{12,\zeta },\;\\ \Xi_{53,\zeta }= & P_{\zeta +1}C_{11,\zeta }+\left(\bar{\alpha }-1\right)L_{\zeta }I_{n},\;\Xi_{54,\zeta }=P_{\zeta +1}\left(I_{1}{\bar{B}}_{\zeta }\right),\;\\ \Xi_{61,\zeta }= & P_{\zeta +1}A_{21,\zeta }+L_{\zeta }A_{22,\zeta },\;\Xi_{62,\zeta }=P_{\zeta +1}B_{21,\zeta }+L_{\zeta }B_{22,\zeta },\;\\ \Xi_{63,\zeta }= & P_{\zeta +1}C_{21,\zeta }+{\tilde{\alpha }}_{\zeta }L_{\zeta }I_{n},\;\\ A_{11,\zeta }= & \left[\begin{array}{cc} {\bar{A}}_{\zeta } & 0\\ \left(1-\bar{\alpha }\right){\bar{D}}_{\zeta } & \bar{\alpha }I_{n} \end{array}\right],\;A_{12,\zeta }=\left[\begin{array}{cc} -\left(1-\bar{\alpha }\right){\bar{D}}_{\zeta } & -\bar{\alpha }I_{n} \end{array}\right],\;\\ B_{11,\zeta }= & \left[\begin{array}{cc} {\bar{C}}_{\zeta } & 0\\ 0 & \left(1-\bar{\alpha }\right){\bar{E}}_{\zeta } \end{array}\right],\;B_{12,\zeta }=\left[\begin{array}{cc} 0 & -\left(1-\bar{\alpha }\right){\bar{E}}_{\zeta } \end{array}\right],\;\\ C_{11,\zeta }= & \left[\begin{array}{c} 0\\ \left(1-\bar{\alpha }\right)I_{n} \end{array}\right],\;A_{21,\zeta }=\left[\begin{array}{cc} 0 & 0\\ -{\tilde{\alpha }}_{\zeta }{\bar{D}}_{\zeta } & {\tilde{\alpha }}_{\zeta }I_{n} \end{array}\right],\;\\ B_{21,\zeta }= & \left[\begin{array}{cc} 0 & 0\\ 0 & -{\tilde{\alpha }}_{\zeta }{\bar{E}}_{\zeta } \end{array}\right],\;A_{22,\zeta }=\left[\begin{array}{cc} {\tilde{\alpha }}_{\zeta }{\bar{D}}_{\zeta } & -{\tilde{\alpha }}_{\zeta }I_{n} \end{array}\right],\;\\ B_{22,\zeta }= & \left[\begin{array}{cc} 0 & {\tilde{\alpha }}_{\zeta }{\bar{E}}_{\zeta } \end{array}\right],\;C_{21,\zeta }=\left[\begin{array}{c} 0\\ -{\tilde{\alpha }}_{\zeta }I_{n} \end{array}\right]. \end{array}$$

Moreover, if the above inequalities are feasible, then the matrix ${\bar{K}}_{\zeta ,\beta_{\zeta }}$ is derived as follows:

(39) $${\bar{K}}_{\zeta ,\beta_{\zeta }}={\left(I_{1}^{T}P_{\zeta +1}I_{1}\right)}^{-1}I_{1}^{T}L_{\zeta }$$

Accordingly, the estimator parameters $K_{ij,\zeta }^{1}$$\left(i=1,\;2,\ldots ,\;n,\;j\in N_{i}\right)$ and $K_{ij,\zeta }^{2}$$\left(i=1,\;2,\ldots ,\;n,\;j\in N_{i}\right)$ can be obtained from (17).

**Proof.**  It is observed that

(40) $$\begin{array}{cc} \; & A_{1,\zeta }=A_{11,\zeta }+I_{1}{\bar{K}}_{\zeta ,\beta_{\zeta }}A_{12,\zeta },\;A_{2,\zeta }=A_{21,\zeta }+I_{1}{\bar{K}}_{\zeta ,\beta_{\zeta }}A_{22,\zeta },\;\\ & B_{1,\zeta }=B_{11,\zeta }+I_{1}{\bar{K}}_{\zeta ,\beta_{\zeta }}B_{12,\zeta },\;B_{2,\zeta }=B_{21,\zeta }+I_{1}{\bar{K}}_{\zeta ,\beta_{\zeta }}B_{22,\zeta },\;\\ \; & C_{1,\zeta }=C_{11,\zeta }+\left(\bar{\alpha }-1\right)I_{1}{\bar{K}}_{\zeta ,\beta_{\zeta }}I_{n},\;C_{2,\zeta }=C_{21,\zeta }+{\tilde{\alpha }}_{\zeta }I_{1}{\bar{K}}_{\zeta ,\beta_{\zeta }}I_{n}. \end{array}$$

Utilizing the Schur Complement Lemma together with the substitution $P_{\zeta +1}I_{1}{\bar{K}}_{\zeta ,\beta_{\zeta }}=L_{\zeta }$, one finds that condition (25) is satisfied if and only if inequality (38) holds. Moreover, by Lemma 2, it is straightforward to check that the required structure ${\bar{K}}_{\zeta }^{1}\in T_{n_{x}\times n_{y}},{\bar{K}}_{\zeta }^{2}\in T_{n_{x}\times n_{y}}$ is indeed guaranteed. This concludes the proof. □

## 4. An Illustrative Example

Consider a wireless sensor network (WSN) consisting of ten nodes and featuring two switched topologies, with the relevant parameters provided below.

The respective adjacency matrixes are shown as follows:

$$\begin{array}{c} A^{\left(1\right)}=\left[\begin{array}{cccccccccc} 1 & 1 & 0 & 0 & 0 & 0 & 0 & 0 & 0 & 0\\ 1 & 1 & 0 & 0 & 0 & 0 & 0 & 0 & 0 & 0\\ 0 & 1 & 1 & 0 & 0 & 0 & 0 & 0 & 0 & 0\\ 0 & 0 & 0 & 1 & 0 & 1 & 0 & 0 & 0 & 0\\ 1 & 0 & 0 & 0 & 1 & 0 & 0 & 0 & 0 & 0\\ 0 & 0 & 0 & 0 & 0 & 1 & 0 & 1 & 0 & 0\\ 0 & 1 & 0 & 0 & 0 & 0 & 1 & 0 & 0 & 0\\ 0 & 1 & 0 & 0 & 0 & 0 & 0 & 1 & 0 & 0\\ 0 & 0 & 0 & 0 & 0 & 1 & 0 & 0 & 1 & 0\\ 1 & 0 & 0 & 0 & 0 & 0 & 0 & 0 & 0 & 1 \end{array}\right],\;A^{\left(2\right)}=\left[\begin{array}{cccccccccc} 1 & 0 & 0 & 1 & 0 & 0 & 0 & 0 & 0 & 0\\ 0 & 1 & 0 & 0 & 0 & 1 & 0 & 0 & 0 & 0\\ 0 & 0 & 1 & 0 & 0 & 0 & 1 & 0 & 0 & 0\\ 1 & 0 & 0 & 1 & 0 & 0 & 0 & 0 & 0 & 0\\ 0 & 0 & 0 & 0 & 1 & 0 & 0 & 0 & 1 & 0\\ 0 & 0 & 1 & 0 & 0 & 1 & 0 & 0 & 0 & 0\\ 0 & 0 & 0 & 0 & 0 & 1 & 1 & 0 & 0 & 0\\ 0 & 0 & 0 & 0 & 0 & 0 & 0 & 1 & 1 & 0\\ 0 & 0 & 1 & 0 & 0 & 0 & 0 & 0 & 1 & 0\\ 0 & 0 & 0 & 0 & 0 & 1 & 0 & 0 & 0 & 1 \end{array}\right]. \end{array}$$

The parameters of the system (1) are given as

$$\begin{array}{cc} \; & A_{\zeta }=\left[\begin{array}{cc} 0.4\sin(\zeta ) & 0.5\cos(2\zeta )\\ 0.8 & 0.3 \end{array}\right],\;B_{\zeta }=\left[\begin{array}{cc} 0.2\sin(2\zeta ) & 0.7\\ 0.5 & 0.5 \end{array}\right],\;C_{\zeta }=\left[\begin{array}{c} 0.8\\ 0.6 \end{array}\right],\;\\ \; & M_{\zeta }=\left[\begin{array}{cc} 0.6 & 0.3 \end{array}\right] \end{array}$$

and the nonlinear function is set to be

$$\begin{array}{cc} \; & f_{x_{\zeta }}=\left[\begin{array}{c} -0.5x_{1,\zeta }+0.4x_{2,\zeta }\\ 0.3x_{1,\zeta }+\frac{\sin(x_{1,\zeta })}{\sqrt{x_{1,\zeta }^{2}+x_{2,\zeta }^{2}+20}} \end{array}\right]. \end{array}$$

where $x_{s,\zeta }\left(s=1,2\right)$ represents the s−th element of the system state $x_{\zeta }$. It is easy to verify that the above nonlinear function $f_{x_{\zeta }}$ satisfies (2) with

$$\begin{array}{cc} \; & N_{1}=\left[\begin{array}{cc} -0.15 & 0.15\\ 0 & 0.30 \end{array}\right],\;N_{2}=\left[\begin{array}{cc} -0.15 & 0.15\\ 0 & 0.40 \end{array}\right]. \end{array}$$

Consider a distributed estimator network consisting of two sensor nodes, with the measurement output parameters given by

$$\begin{array}{cc} \; & D_{1,\zeta }=\left[\begin{array}{cc} -0.02\sin(2\zeta ) & 0.3 \end{array}\right],\;D_{2,\zeta }=\left[\begin{array}{cc} 0.03\sin(2\zeta ) & 0.4 \end{array}\right],\\ \; & D_{3,\zeta }=\left[\begin{array}{cc} 0.03\sin(2\zeta ) & -0.2 \end{array}\right],\;D_{4,\zeta }=\left[\begin{array}{cc} 0.03\sin(\zeta ) & 0.3 \end{array}\right],\\ \; & D_{5,\zeta }=\left[\begin{array}{cc} 0.04\sin(2.5\zeta ) & -0.2 \end{array}\right],\;D_{6,\zeta }=\left[\begin{array}{cc} 0.02\sin(2\zeta ) & 0.4 \end{array}\right],\\ \; & D_{7,\zeta }=\left[\begin{array}{cc} 0.01\sin(2\zeta ) & -0.3 \end{array}\right],\;D_{8,\zeta }=\left[\begin{array}{cc} 0.05\sin(2\zeta ) & 0.1 \end{array}\right],\\ \; & D_{9,\zeta }=\left[\begin{array}{cc} 0.04\sin(\zeta ) & -0.2 \end{array}\right],\;D_{10,\zeta }=\left[\begin{array}{cc} 0.02\sin(3\zeta ) & 0.3 \end{array}\right],\\ \; & E_{1,\zeta }=0.7,\;E_{2,\zeta }=0.8,\;E_{3,\zeta }=0.6,\;E_{4,\zeta }=0.6,\;E_{5,\zeta }=0.7,\\ \; & E_{6,\zeta }=0.7,\;E_{7,\zeta }=0.7,\;E_{8,\zeta }=0.8,\;E_{9,\zeta }=0.4,\;E_{10,\zeta }=0.8,\\ \; & \rho =0.3,\;\bar{\alpha }=0.75,\;\bar{\beta }=0.7,\;\varepsilon =0.02. \end{array}$$

The event generator parameters are chosen as $\Omega_{1,\zeta }=\Omega_{2,\zeta }=\Omega_{3,\zeta }=I$, with initial thresholds set to $\vartheta_{1}=0.25$, $\vartheta_{2}=0.2$, $\vartheta_{3}=0.3$, $\vartheta_{4}=0.3$, $\vartheta_{5}=0.25$, $\vartheta_{6}=0.33$, $\vartheta_{7}=0.28$, $\vartheta_{8}=0.22$, $\vartheta_{9}=0.2$ and $\vartheta_{10}=0.4$. The prescribed average $H_{\infty }$ disturbance attenuation level is taken as γ=0.9. The positive definite matrices are selected as $S_{i}=\mathrm{diag}\left\{2,2\right\}$ for i=1,2,3, and the initial values are given by $x_{0}={\left[\begin{array}{cc} 0.25 & 0.45 \end{array}\right]}^{T}$, ${\hat{x}}_{1,0}={\left[\begin{array}{cc} 0.15 & 0.25 \end{array}\right]}^{T}$, ${\hat{x}}_{2,0}={\left[\begin{array}{cc} 0.2 & 0.15 \end{array}\right]}^{T}$, ${\hat{x}}_{3,0}={\left[\begin{array}{cc} 0.2 & 0.25 \end{array}\right]}^{T}$, ${\hat{x}}_{4,0}={\left[\begin{array}{cc} 0.25 & 0.2 \end{array}\right]}^{T}$, ${\hat{x}}_{5,0}={\left[\begin{array}{cc} 0.3 & 0.25 \end{array}\right]}^{T}$, ${\hat{x}}_{6,0}={\left[\begin{array}{cc} 0.25 & 0.25 \end{array}\right]}^{T}$, ${\hat{x}}_{7,0}={\left[\begin{array}{cc} 0.35 & 0.3 \end{array}\right]}^{T}$, ${\hat{x}}_{8,0}={\left[\begin{array}{cc} 0.3 & 0.15 \end{array}\right]}^{T}$, ${\hat{x}}_{9,0}={\left[\begin{array}{cc} 0.4 & 0.15 \end{array}\right]}^{T}$ and ${\hat{x}}_{10,0}={\left[\begin{array}{cc} 0.2 & 0.2 \end{array}\right]}^{T}$. One can easily verify that the positive definite matrix $P_{0}=1.2I$ meets the initial requirement (37). By solving the RLMIs (38) using Matlab R2014a the distributed estimator gains are obtained.

**Figure 1:**
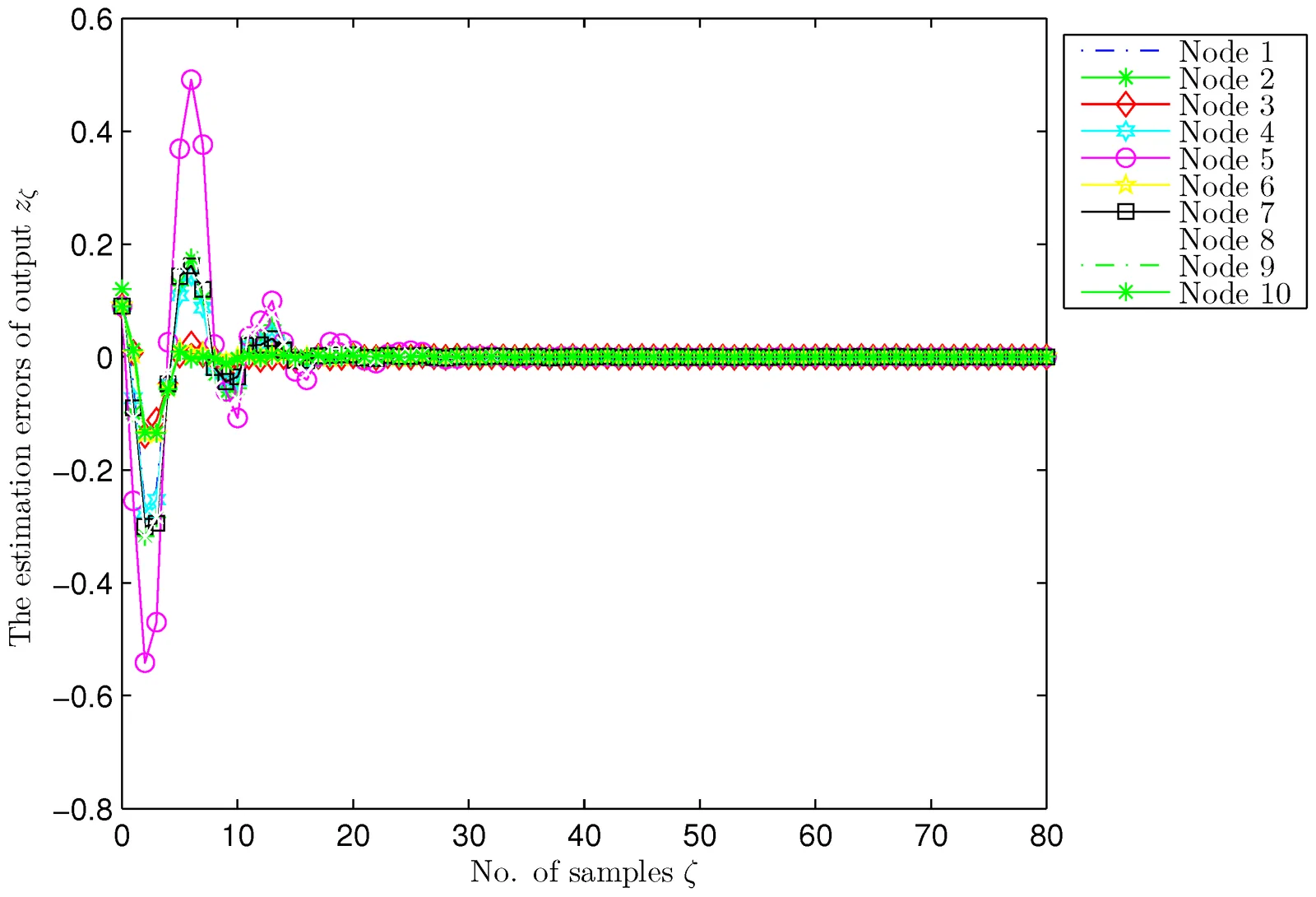
The estimation errors of the output z(ζ).

**Figure 2:**
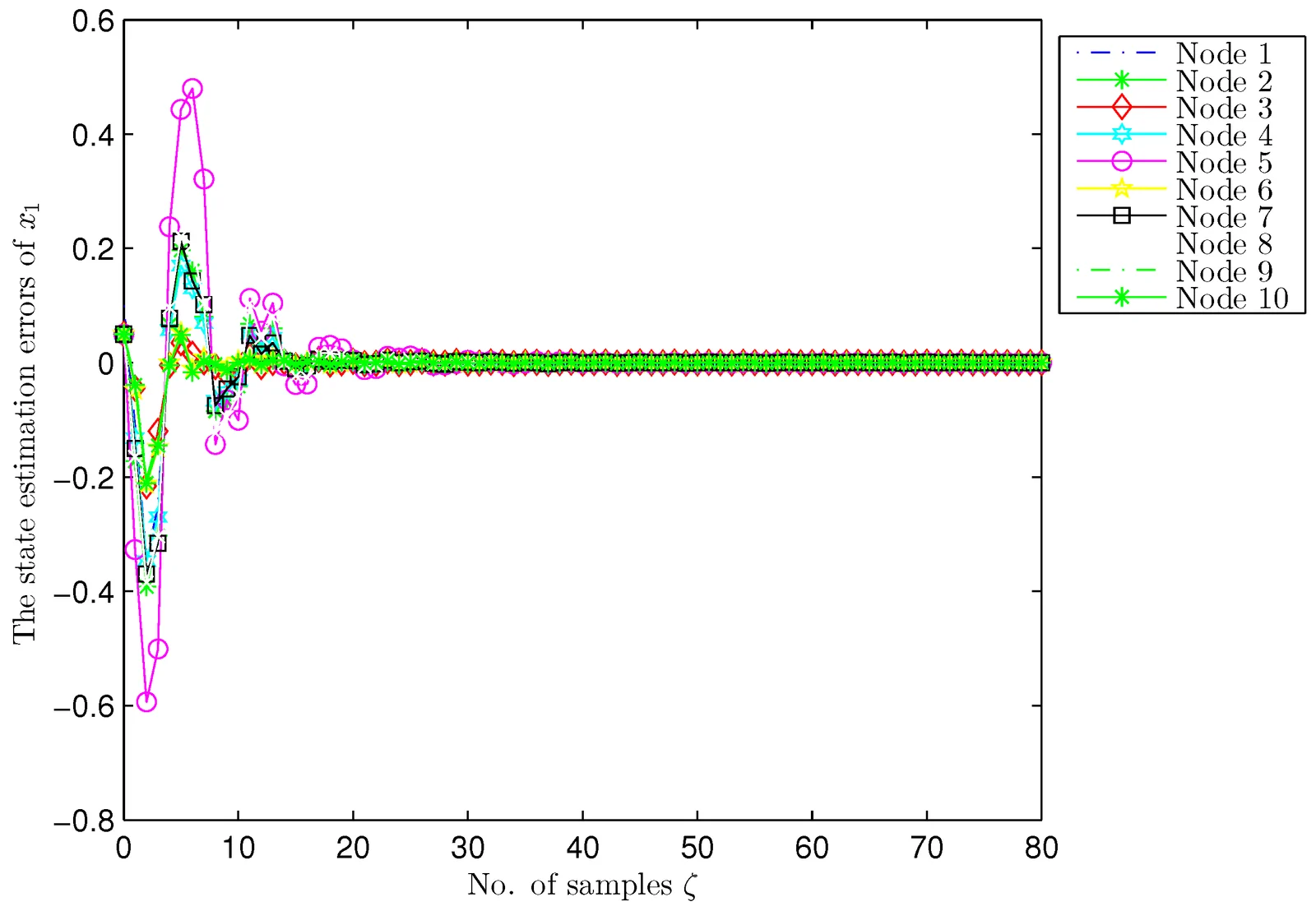
The state estimation errors of $x_{1}\left(\zeta \right)$.

**Figure 3:**
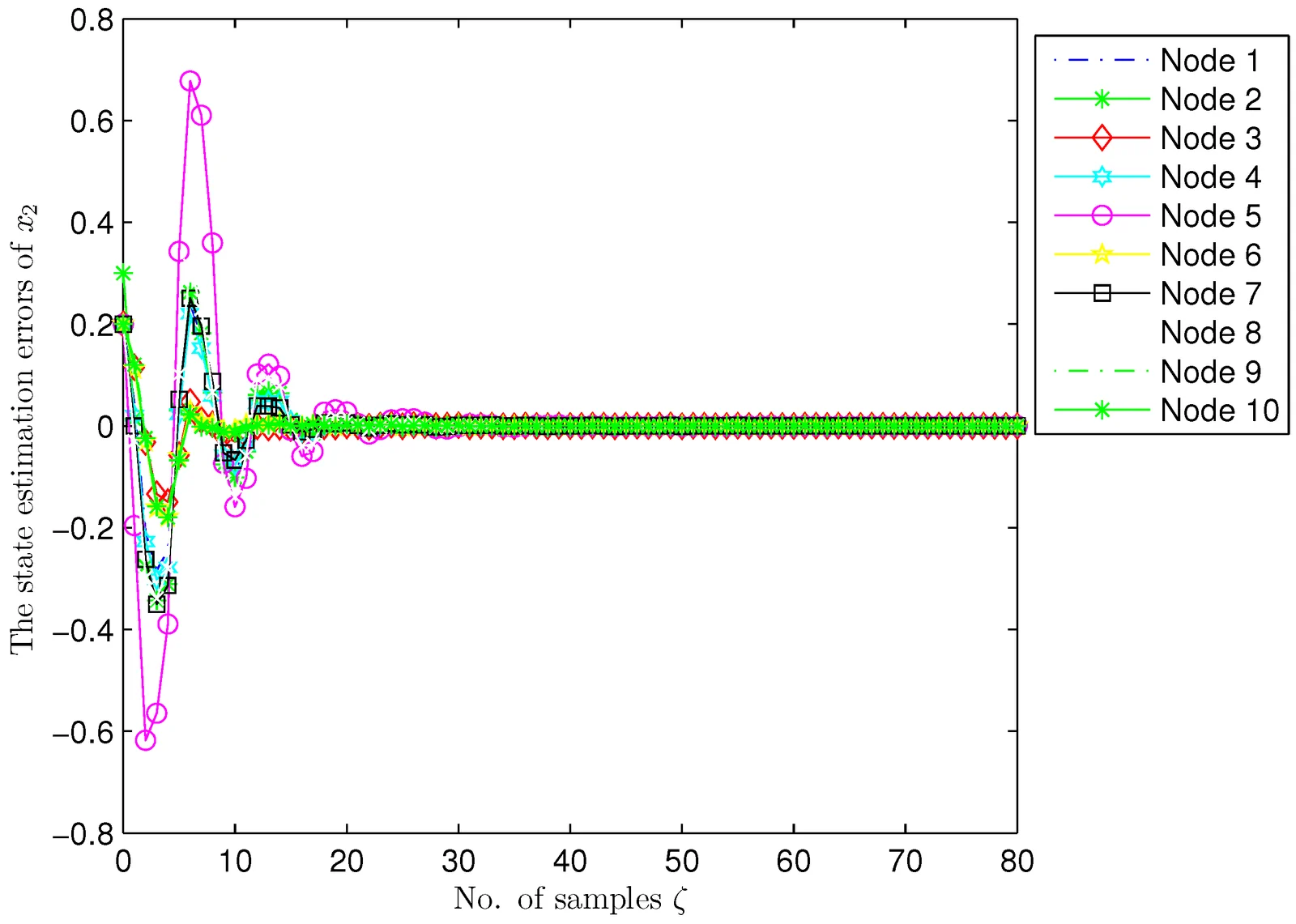
The state estimation errors of $x_{2}\left(\zeta \right)$.

**Figure 4:**
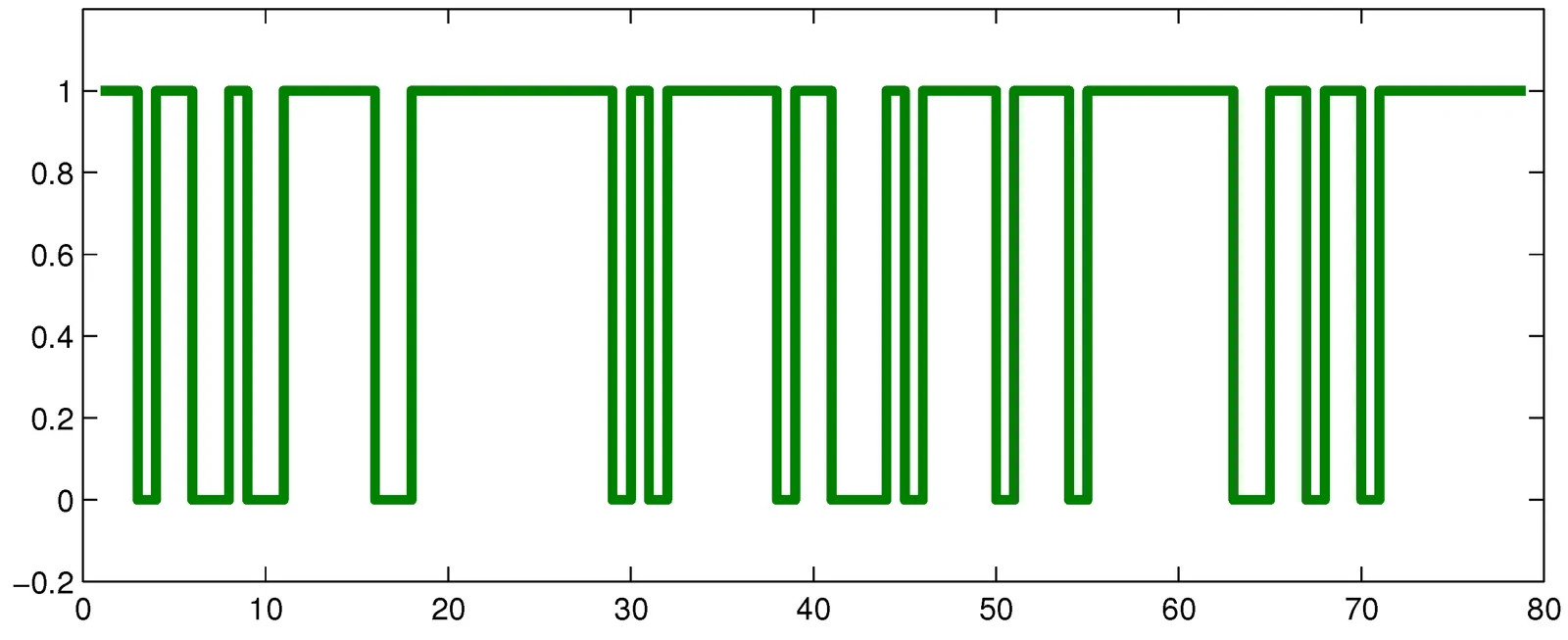
The topology switching frequency.

**Figure 5:**
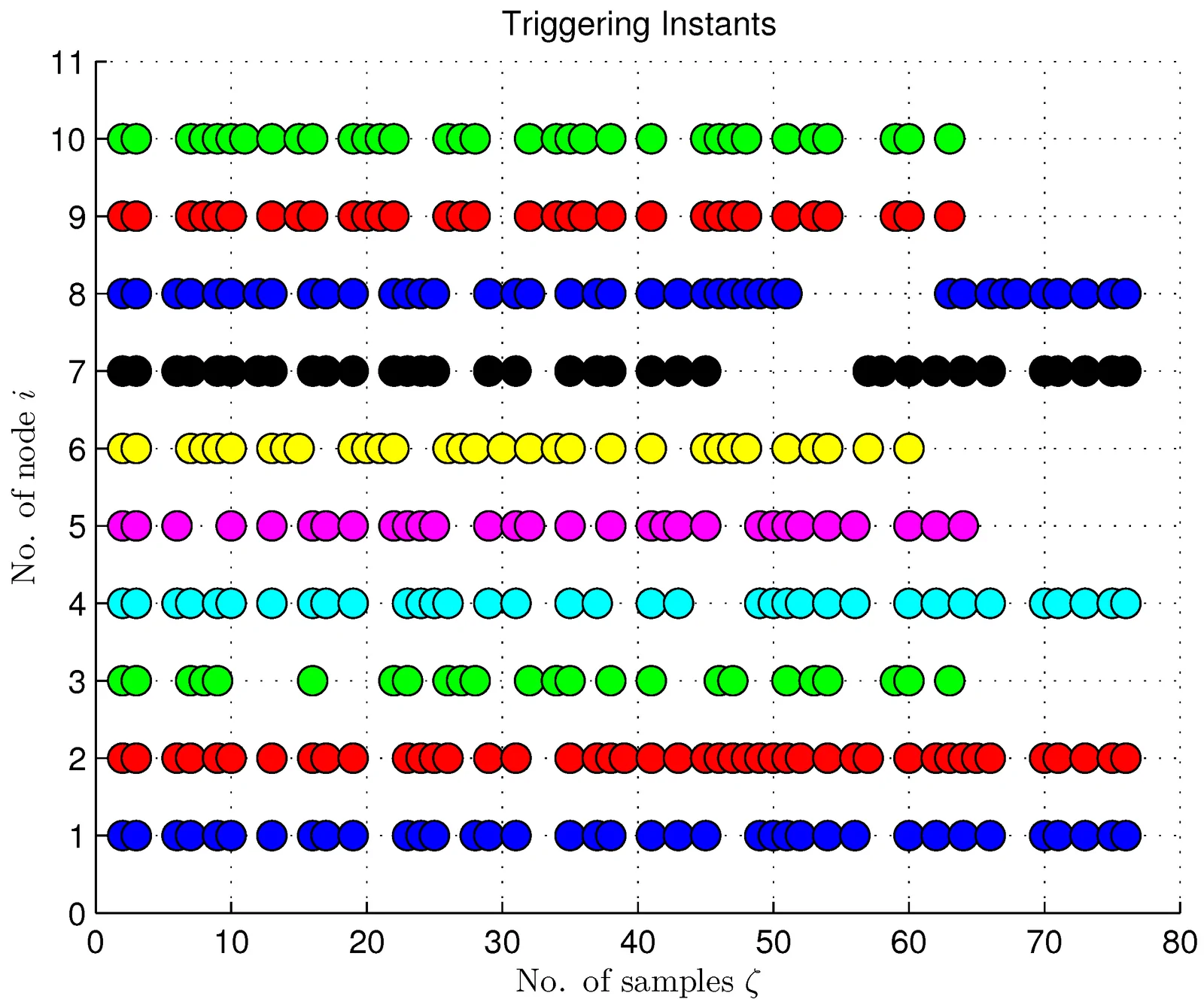
The adaptive event-triggering instants.

**Figure 6:**
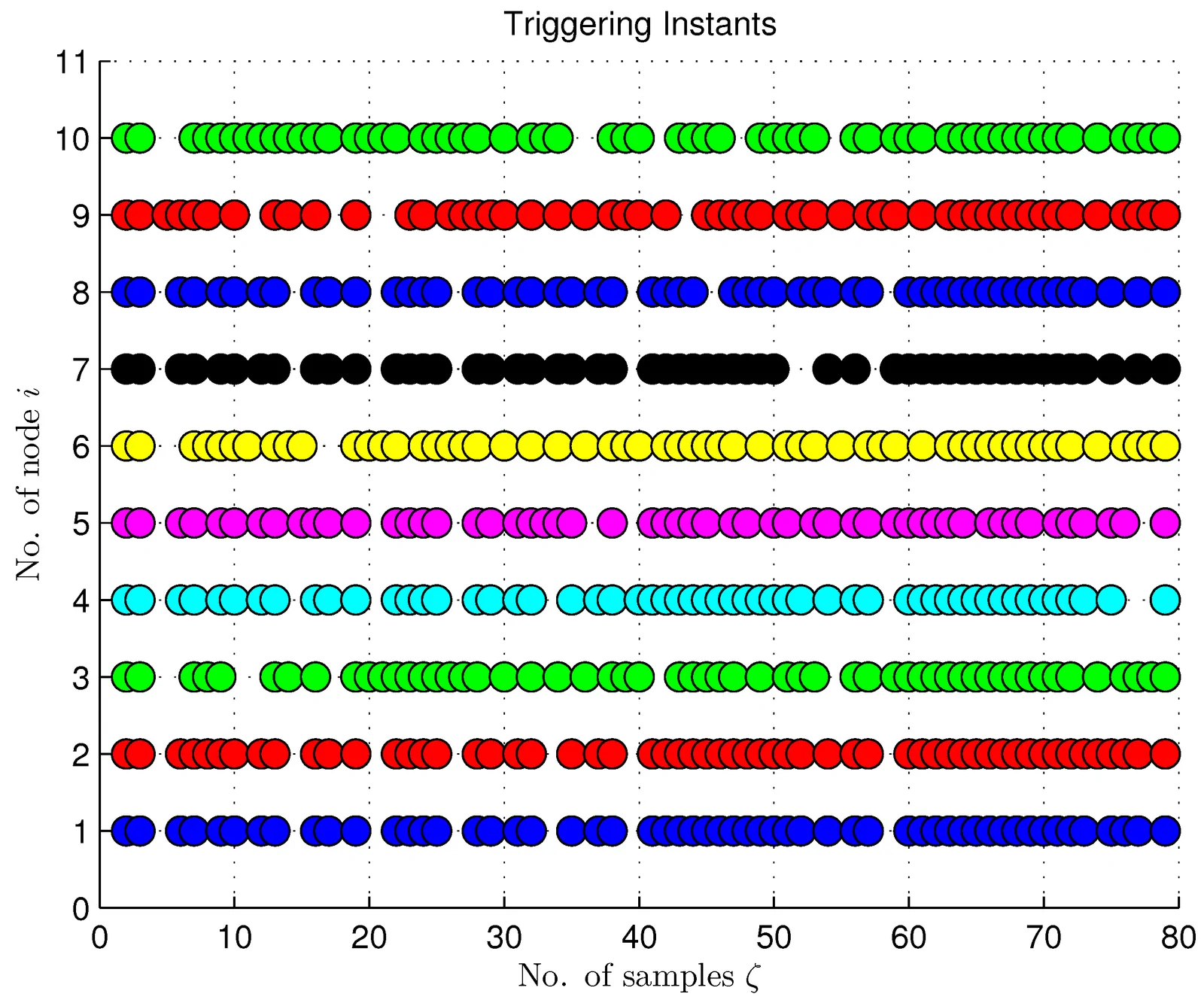
The traditional event-triggering instants.

The external disturbances are chosen as $w_{\zeta }=\frac{0.5}{{\left(\zeta +3\right)}^{1.5}}\sin\left(2\zeta \right)$ and $v_{\zeta }=\frac{0.8}{{\left(\zeta +1\right)}^{2}}\sin\left(\zeta \right)$. The simulation outcomes are illustrated in Figure 1, Figure 2, Figure 3, Figure 4, Figure 5 and Figure 6. Specifically, Figure 1 displays the estimation errors of $z_{\zeta }$ at each sensor node, Figure 2 presents the state estimation errors for $x_{1,\zeta }$, Figure 3 shows the state estimation errors for $x_{2,\zeta }$, Figure 4 gives the topology switching sequence, Figure 5 plots the adaptive event-triggering instants and Figure 6 plots the traditional event-triggering instants. These simulation results confirm the effectiveness of the proposed event-triggered distributed state estimation scheme.

## 5. Conclusions

This paper investigates the distributed estimation problem for discrete time-varying systems over sensor networks subject to replay attacks, where an adaptive event-triggered communication strategy is adopted. A dedicated distributed estimator is designed for such time-varying networked systems under attack conditions. By integrating the adaptive event-triggered scheme, the communication load for transmitting data from sensor nodes to the estimator is significantly reduced. A design approach for the distributed estimator is developed to guarantee that the resulting augmented estimation error dynamics satisfy the prescribed average $H_{\infty }$ performance constraint. A numerical example is presented to demonstrate the effectiveness of the proposed estimation algorithm. The theoretical results derived in this work may potentially be extended to other types of networked systems.

## Data Availability

The original contributions presented in this study are included in the article. Further inquiries can be directed to the corresponding author.

## Abbreviations

The following abbreviations are used in this manuscript:

WSNs	Wireless sensor networks
ET	Event-triggered
DoS	Denial-of-service
RLMIs	Recursive linear matrix equalities
